# Fever-Associated Supraventricular Tachycardia after 4CMenB Vaccination in an Infant

**DOI:** 10.1155/2019/4591964

**Published:** 2019-09-22

**Authors:** Erdem Gönüllü, Burcu Topçu, Naci Öner, Ahmet Soysal, Metin Karaböcüoğlu

**Affiliations:** ^1^Memorial Ataşehir Hospital, Department of Pediatrics, 34758 Istanbul, Turkey; ^2^Memorial Ataşehir Hospital, Department of Pediatric Cardiology, 34758 Istanbul, Turkey; ^3^Memorial Ataşehir Hospital, Department of Pediatric Infectious Diseases, 34758 Istanbul, Turkey

## Abstract

Meningococcal serogroup B vaccine 4CMenB (Bexsero) is a new four-component protein vaccine developed to prevent *Neisseria meningitidis* serogroup B infections. *Case*. We report a girl with fever and supraventricular tachycardia (SVT) 6–8 hours after the second dose of 4CMenB. SVT was unresponsive to the first dose of adenosine but terminated after the fourth dose of adenosine. During three months of follow-up, she was free of further SVT attacks. *Conclusion*. This is the first report of ECG-proven SVT after 4CMenB vaccination. Even if fever is coexistent, SVT should be considered after persistent tachycardia and 4CMenB vaccination.

## 1. Introduction

*Neisseria meningitidis*, a Gram-negative diplococcus and an obligate human pathogen, is the leading cause of bacterial meningitis and septicemia in children worldwide [1]. The four-component meningococcal serogroup B vaccine 4CMenB (Bexsero®, GSK) is a vaccine against this serogroup and has been approved by licensing authorities in Europe, the USA, Canada, and Australia [2].

4CMenB vaccine was introduced to the UK National Immunization Programme (NIP) in September 2015 with a schedule primary dose given at 2 and 4 months of age and a booster dose given at 12 months of age together with other routine childhood vaccines. In Turkey, 4CMenB was licensed in November 2018 for children above 2 months of age with a 3 + 1 schedule primary dose given at 2, 4, and 6 months at age and a booster dose given after 12 months of age alone or together with other routine childhood vaccines. The vaccine was not introduced in the National Immunization Programme (NIP) in Turkey. The 4CMenB vaccine was associated with higher frequencies of local reactions and fever than other routine infant vaccines, particularly when given concomitantly with other vaccines, which were mostly transient and of mild-to-moderate severity [3]. For this reason, many countries recommended prophylactic paracetamol usage before 4CMenB vaccination for children under 24 months of age [3]. The most commonly reported serious adverse events were febrile convulsions, arthritis, and Kawasaki disease [4]. We herein report a case of supraventricular tachycardia (SVT) in a girl 6–8 hours after the second dose of the 4CMenB injection.

## 2. Case

**Figure 1:**
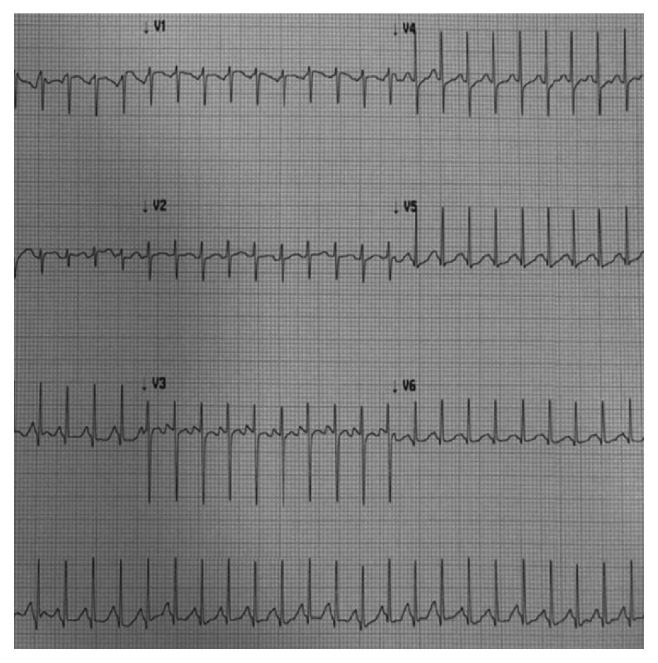
Electrocardiogram showing the supraventricular tachycardia.

A previously healthy 11-month-old girl was admitted to the pediatric emergency room with fever (38.3°C) and vomiting 6–8 hours after the second dose of the 4CMenB vaccine on the left lateral thigh, although she had received prophylactic paracetamol before vaccination. Her vomiting was not massive and did not repeat in the hospital. Her physical examination revealed irritability and tachycardia with good perfusion. She was not agitated, and the injection site was not painful. Her heart rate was over 200 per minute in rest without any cardiac murmur or additional heart sounds. Laboratory workup results were as follows: hemoglobin 12.9 g/dL, white blood cell 5170/*μ*L, platelet count 380,000/*μ*L, CRP 0.9 mg/L (*N* < 7.9), and procalcitonin 0.01 ng/mL (*N*: <0.5), respectively. Influenza A and B, respiratory syncytial virus, adenovirus nasopharyngeal swab antigen assays, and streptococcal pharyngeal swab antigen test were all negative. Her plasma glucose was 90 mg/dl, and other biochemistry values were normal but creatinine kinase-MB was elevated (5.2 ng/ml, N: 0–3.61). After verifying SVT with narrow QRS complex without p waves (Figure 1), vagal stimulatory maneuvers were applied but her heart rate did not become normal. Then, rapid adenosine infusion protocol with 100 *μ*g/kg was injected via a large antecubital venous catheter. Meanwhile, she was hospitalized and monitored, but SVT did not reverse after the first adenosine injection. Then, second, third, and fourth adenosine injections were given with dosage increment to a maximum dose of adenosine (350 *μ*g/kg, total 3000 *μ*g). Her heart rate was converted after 5 seconds to normal sinus rhythm after infusion of the fourth dose of adenosine, total 2 hours of arrival. Then, oral propranolol (3 mg/kg/day, every 8 hours) maintenance treatment was started. Echocardiography revealed normal cardiac anatomy with slightly decreased left ventricle function (EF: 48%; left ventricle end-diastolic volume was 32.2 mm) during SVT and normalized after two days. She was followed for 3 months, and no recurrence of SVT was detected.

## 3. Discussion

The novel multicomponent meningococcal vaccine 4CMenB has previously been licensed by several licensing authorities worldwide. As with any new vaccine, knowledge of the safety profile of the vaccine is limited to the size of the clinical trials. Data from 4CMenB clinical trials with more than 6000 children suggest that the frequency of local and systemic reactions would be expected to substantially increase if 4CMenB was given with other routine infant immunizations [2]. In a recent prospective cohort study of routine 4CMenB recipient infants in the United Kingdom, after more than 3 million doses were given to about 1.29 million infants over 20 months across the country, 902 of them reported for vaccine-related adverse events. Three hundred sixty-four (40%) were of fever, 55 (6%) of seizures, 366 (41%) of skin reactions, three (<1%) of Kawasaki disease, and five (<1%) of 902 reports had a fatal outcome including one report of sudden infant death syndrome, one report of sudden unexplained death, and three reports of death, all within 3 days of receiving 4CMenB alongside routine infant vaccination [3]. To the best of our knowledge, there is currently no report about SVT soon after 4CMenB vaccination.

Supraventricular tachycardia, a common acquired cardiac condition involving a heterogeneous grouping of arrhythmias, has been observed in 0.1–0.4% of the pediatric population, with a prevalence rate twice as high in females as well as patients of various ethnic and age differences [5]. Even subclinical, an inflammatory response in infancy may cause SVT [6]. The majority of SVTs are due to reciprocating atrioventricular (AV) tachycardia rather than rapid firing of a single focus in the atria (ectopic atrial tachycardia) or in the AV node (ectopic nodal tachycardia). AV reentrant or reciprocating tachycardia is not only the most common mechanism of SVT but also the most common tachyarrhythmia seen in the pediatric age group. 4CMenB and the fever that it causes may have triggered the reentry mechanism or may have annoyed ectopic focus causing SVT in this infant. Propranolol has been shown to be both safe and effective in neonates and infants, even at high doses, and propranolol is favored by pediatric electrophysiologists for the chronic management of SVT [7].

In our case, a febrile onset SVT episode was observed after the second dose of 4CMenB in the 11-month-old girl, although prophylactic paracetamol was given in accordance with the recommendations. Her physical examination and laboratory workup with viral antigens for common upper respiratory viral antigens were negative. She had no heart disease damaging the cardiac pathways including myocarditis, dilated cardiomyopathies, and congenital heart diseases such as Ebstein's anomaly, single ventricle, and congenitally corrected transposition of the great arteries. She did not have any accessory pathways as the Wolff–Parkinson–White syndrome. The aforementioned diseases render the patient more susceptible to SVT. Her clinical course was uncomplicated, and she was discharged with oral propranolol therapy (3 mg/kg/d, oral, every eight hours) after 24 hours without complication. Following the family's request, the last dose of 4CMenB was not given.

## 4. Conclusion

We would like to emphasize that prophylactic paracetamol may reduce fever and fever-associated SVT after 4CMenB vaccination.
